# New species of *Medetera* from Inner Mongolia, China (Diptera, Dolichopodidae, Medeterinae)

**DOI:** 10.3897/zookeys.604.8377

**Published:** 2016-07-11

**Authors:** Chufei Tang, Ning Wang, Ding Yang

**Affiliations:** 1 Department of Entomology, College of Plant Protection, China Agricultural University, Beijing 100193, China; 2 Institute of Grassland Research, Chinese Academy of Agricultural Sciences, Hohhot, Inner Mongolia 010010, China

**Keywords:** Diptera, Dolichopodidae, Inner Mongolia, Medeterinae, Medetera, new species

## Abstract

Only three species of *Medetera* Fischer von Waldheim were known from Inner Mongolia. Here the following ten new species of *Medetera*, of which three species belong to *Medetera apicalis* group and seven belong to *Medetera diadema*-*veles* group, are added to the fauna of Inner Mongolia: *Medetera albens* **sp. n.**, *Medetera bisetifera* **sp. n.**, *Medetera flava* **sp. n.**, *Medetera ganshuiensis* **sp. n.**, *Medetera lihuae* **sp. n.**, and *Medetera transformata* **sp. n.**, *Medetera triseta* **sp. n.**, *Medetera shiae* **sp. n.**, *Medetera shuimogouensis* **sp. n.**, and *Medetera xiquegouensis* **sp. n.** A key to the species of *Medetera* from Palaearctic China is provided.

## Introduction

*Medetera* Fischer von Waldheim is a large genus with nearly 360 known species around the world (Negrobov and Naglis 2015, Tang et al. 2015). Most of them are small, dark metallic green or even black, with thin pollinosity. The genus can be separated from other genera of Medeterinae by the following features: first flagellomere rounded with apical or subapical arista, occiput concave, proboscis heavily sclerotized, mesoscutum strongly flattened, vein M_1+2_ strongly convergent to vein R_4+5_ beyond discal crossvein, and the large and pedunculate male genitalia tightly flexed to ventral surface of abdomen (Masunaga and Saigusa 1998, Bickel 1985, 1987). *Medetera apicalis* species group and *Medetera diadema*-*veles* species group can be separated into clades by molecular evidence (Pollet et al. 2011). The species diversity of *Medetera* is extremely rich in the Palaearctic region, which has 178 described species and comprises more than half of the whole genus (Yang et al. 2006, Naglis and Negrobov 2015). The latest comprehensive taxonomic work on the Palaearctic species is the revision of the subfamily Medeterinae by Negrobov and Stackelberg (1971-1977). Since then 27 new Palaearctic species of *Medetera* have been described: one species from Great Britain (Allen 1976), one from Poland (Negrobov and Capecki 1977), six from Russia (Negrobov 1979, Negrobov and Golubtzov 1991, Negrobov and Naglis 2015), one from Spain (Rampini and Canzoneri 1979), four from Japan (Masunaga and Saigusa 1998), one from China (Yang 1999), one from Morocco (Grichanov and Vikhrev 2009), one from Tunisia (Grichanov 2010), three from Turkey (Naglis 2013), seven from Switzerland (Naglis and Negrobov 2014a, b), and one species from Mongolia (Negrobov and Naglis 2015).

Species of *Medetera* have some interesting behavioral characteristics such as their stance on tree trunks or walls which resembles that of woodpeckers, in fact they have been referred to as “woodpecker flies” (Bickel 1985). *Medetera* species are predators of bark beetles (Coleoptera: Curculionidae) and some other pests like aphids, psychodids and myriapods (Beaver 1966, Ulrich 2005). Ulrich (2005) also mentioned that the strongly developed labium of *Medetera* is able to crack hard parts, for example the exoskeleton of prey, into small fragments. It is suggested that the genus may have potential use in biological control. Most species of Medeterinae recorded, including *Medetera*, occur in dry and relatively cold environments. Species from tropical areas are relatively poorly described. However, in China the reverse is true with only four species of *Medetera* recorded in the Palaearctic but 20 species recorded in the Oriental (Yang et al. 2011, Tang et al. 2015), which means more species are known in the Oriental region than the Palaearctic region. It is suspected that the circumstance is due to the incomplete investigation of Palaearctic China.

Inner Mongolia is a large province of China, which stretches from northeast to northwest of China. Most of its international border is with Mongolia while a small portion is with Russia. The temperate continental climate leads to the specific vegetation. The plain is cold and arid, usually only grass can grow and herding of goats is common. The environments where we collected all the specimens are in small moist areas surrounded by dry land, or dry land near rivers. Specimens of *Medetera* were found in and among tall grasses like *Stipa*. Here ten new species are added to *Medetera* from Inner Mongolia, of which seven belong to *Medetera diadema*-*veles* group, including *Medetera flava* sp. n., *Medetera ganshuiensis* sp. n., *Medetera lihuae* sp. n., *Medetera shiae* sp. n., *Medetera shuimogouensis* sp. n., *Medetera transformata* sp. n. and *Medetera xiquegouensis* sp. n.; three belong to *Medetera apicalis* group, including *Medetera albens* sp. n., *Medetera bisetifera* sp. n. and *Medetera triseta* sp. n. It is the first report of *Medetera apicalis* group in Palaearctic China. A key to species of *Medetera* in Palaearctic China is provided.

## Material and methods

The specimens on which this study is based were collected from Inner Mongolia from 2010 to 2014 by sweeping nets in grassland. All specimens are deposited in the Entomological Museum of China Agricultural University
(CAU), Beijing. Morphological terminology for adult structures mainly follows McAlpine (1981). Terms for the structures of the male genitalia follow Cumming and Wood (2009). The following abbreviations are used: acr = acrostichal bristle (s), ad = anterodorsal bristle (s), av = anteroventral bristle (s), dc = dorsocentral bristle (s), pd = posterodorsal bristle (s), pp = propleuron, pv = posteroventral bristle (s), v = ventral bristle (s), sa = supraalar bristle (s), sc = scutellars, CuAx ratio = length of crossvein dm–cu / length of distal portion of vein CuA, LI = fore leg, LII = mid leg, LIII = hind leg.

## Taxonomy

### Key to species (males) of *Medetera* in Palaearctic China

**Table:** 

1	Epandrium almost as long as wide, surstylus short and wide; hypandrium with two lateral hollows at base	***Medetera tuberculata* Negrobov**
–	Epandrium distinctly longer than wide, at least 1.5 times longer than wide; surstylus long and thin	**2**
2	Male hind tarsomere 1 with distinct basal anteroventral tooth; male genitalia pyriform, basally inflated; epandrial lobes fused at least basally; epandrial bristle reduced or lost; hypandrium and phallus elongate, narrow, tapering (Fig. 17) (***diadema*-*veles* group**) (Bickel 1985)	**3**
–	Male hind tarsomere 1 without basal tooth; male genitalia subrectangular; epandrial lobes with bases separated; epandrial bristle well-developed; hypandrium elongate, subrectangular, often basally “clasping” the phallus, and often held out at an angle from male genitalia (Fig. 12) (***apicalis* group**) (Bickel 1985)	**12**
3	Cercus without wide blade-like bristle apically (Figs 25, 29)	**4**
–	Cercus with one wide blade-like bristle apically	**9**
4	Cercus without distinct bristle apically (Fig. 25)	***Medetera shuimogouensis* sp. n.**
–	Cercus with claw-like bristles(s) apically	**5**
5	M_1+2_ and R_4+5_ both arched towards R_2+3_ (Fig. 10)	***Medetera xiquegouensis* sp. n.**
–	M_1+2_ and R_4+5_ normal	**6**
6	Hind tarsomere 1 with an incision and one small spur at base	***Medetera micacea* Loew**
–	Hind tarsomere 1 without incision at base	**7**
7	One sa; cercus ventrally with one process near apex	***Medetera latipennis* Negrobov**
–	Two sa; cercus various	**8**
8	Antennae brown except scape yellow; basolateral bristle of epandrial lobe feather-like at apical half (Yang et al. 2010, fig. 258); hind 2^nd^ tarsomere three times length of tarsomere 1	***Medetera plumbella* Meigen**
–	Antennae wholly black; basolateral bristle of epandrial lobe wholly feather-like (Fig. 33); hind 2^nd^ tarsomere 2.5 times length of tarsomere 1	***Medetera flava* sp. n.**
9	Dorsal surstylus U-shaped with two arms that are similar in shape	**10**
–	Dorsal surstylus U-shaped with two asymmetrical arms, with dorsal arm three times wider than ventral arm (Fig. 27)	***Medetera transformata* sp. n.**
10	Arms of dorsal surstylus sharp apically; ventral surstylus narrowed towards tip, with two long strong lateral bristles at apical 1/5 (Fig. 19)	***Medetera ganshuiensis* sp. n.**
–	Arms of dorsal surstylus round apically; ventral surstylus simple, only with some simple apical bristles	**11**
11	Four uniseriate acr; CuAx ratio 1.00 (Fig. 6); cercus nearly rectangular, six times longer than wide (Fig. 21)	***Medetera lihuae* sp. n.**
–	Eight biseriate acr; CuAx ratio 0.61 (Fig. 7); Cercus strip-like, narrowed towards tip, base dilated, three times longer than the widest point (Fig. 23)	***Medetera shiae* sp. n.**
12	Tibiae totally black; arista turning black to pale yellow from base to tip (Fig. 1); apical bristle of cercus somewhat claw-like (Fig. 12)	***Medetera albens* sp. n.**
–	Tibiae yellow or mainly yellow except basal and apical 1/3 of fore tibia black; arista wholly black; apical bristle of cercus various	**13**
13	CuAx ratio 0.30 (Fig. 2); cercus strip-like, 6 times longer than wide, apical bristle normal (Fig. 14); phallus with distinct preapical lateral wings in ventral view (Fig. 31)	***Medetera bisetifera* sp. n.**
–	CuAx ratio 0.67 (Fig. 3); cercus strip-like, 2.5 times longer than wide, apical bristle blade-like (Fig. 15); phallus normal (Fig. 32)	***Medetera triseta* sp. n.**

**Figures 1–6.**
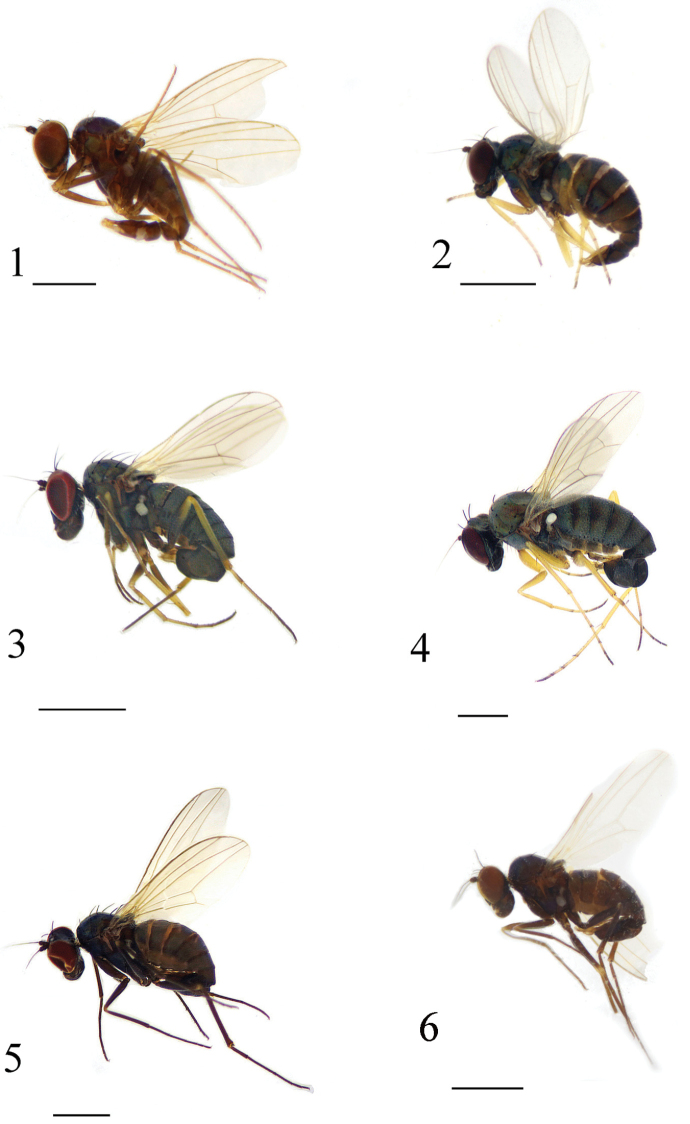
Habitus, lateral view (male). **1** *Medetera albens* sp. n. **2** *Medetera bisetifera* sp. n. **3** *Medetera triseta* sp. n. **4** *Medetera flava* sp. n. **5** *Medetera ganshuiensis* sp. n. **6** *Medetera lihuae* sp. n. Scale bars: 1 mm.

**Figures 7–10.**
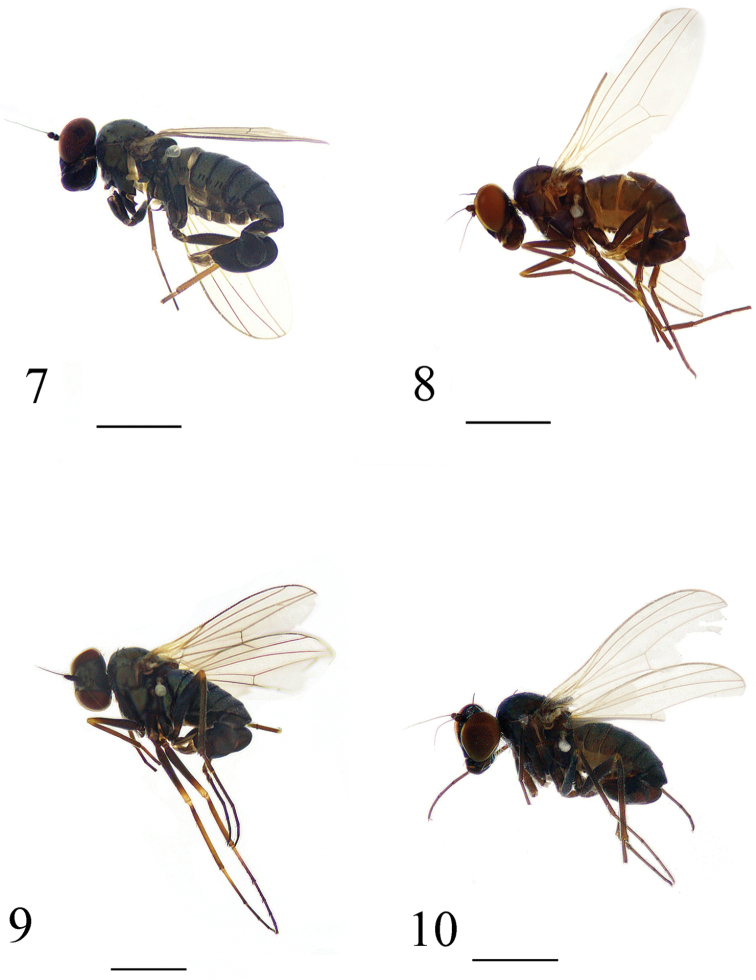
Habitus, lateral view (male). **7** *Medetera shiae* sp. n. **8** *Medetera shuimogouensis* sp. n. **9** *Medetera transformata* sp. n. **10** *Medetera xiquegouensis* sp. n. Scale bars: 1 mm.

**Figures 11–12.**
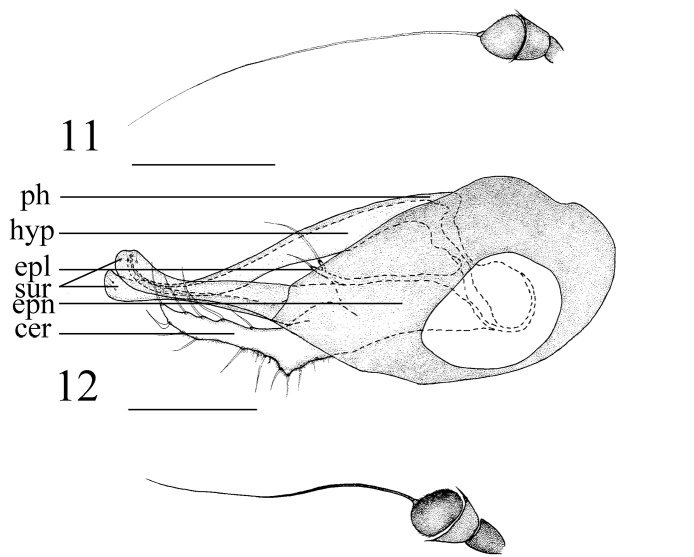
*Medetera albens* sp. n., male. **11** Antenna **12** genitalia, lateral view. Abbreviations: epl = epandrial lobe, epn = epandrium, hyp = hypandrium, ph = phallus, sur = surstylus, cer = cercus. Scale bar: 0.2 mm

**Figures 13–14.**
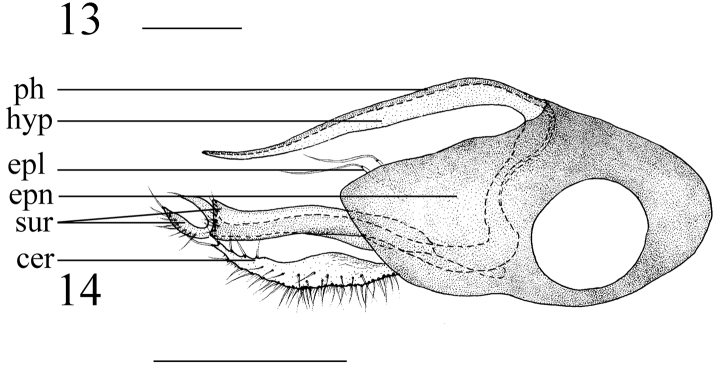
**13** Antenna **14** genitalia, lateral view. Abbreviations: epl = epandrial lobe, epn = epandrium, hyp = hypandrium, ph = phallus, sur = surstylus, cer = cercus. Scale bar: 0.2 mm

**Figures 15.**
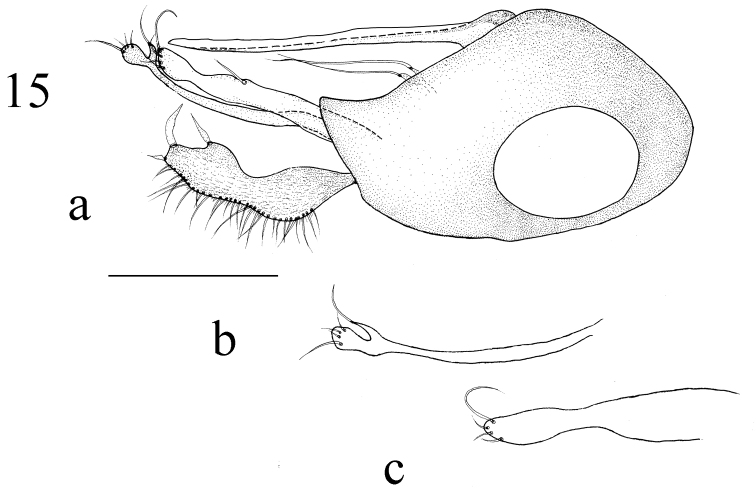
*Medetera triseta* sp. n., male. a genitalia, lateral view **b** dorsal surstylus, lateral view **c** ventral surstylus, lateral view. Scale bar: 0.2 mm.

**Figures 16–17.**
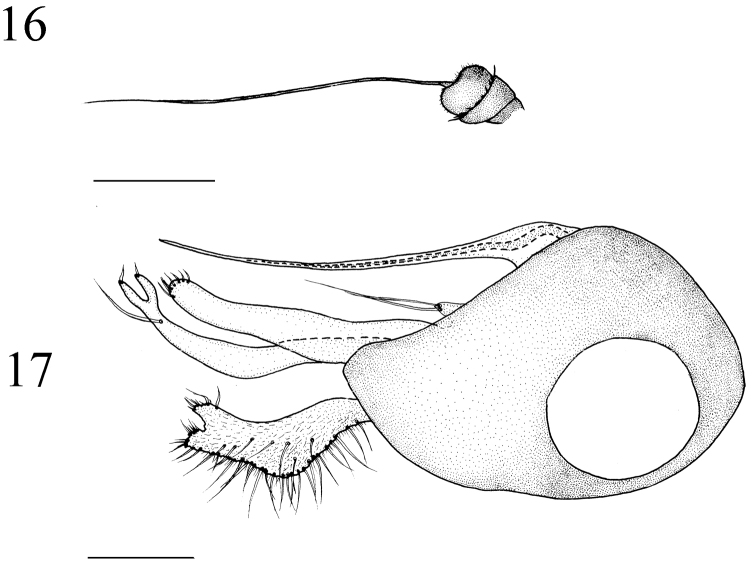
*Medetera flava* sp. n., male. **16**. Antenna **17** genitalia, lateral view. Scale bar = 0.2 mm.

**Figures 18–19.**
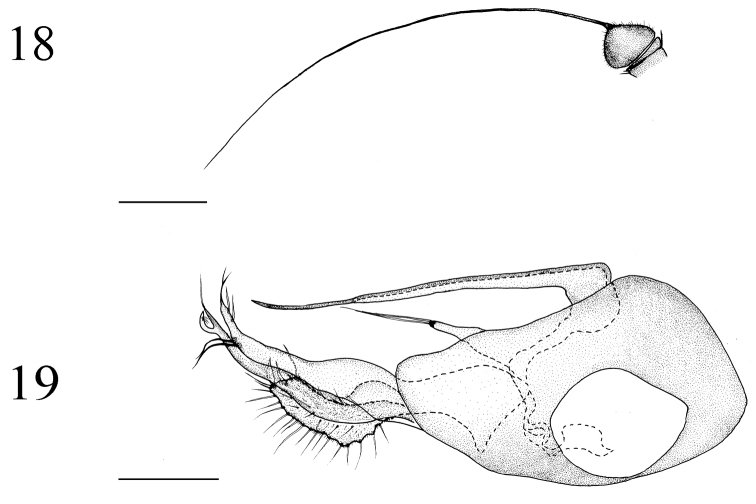
*Medetera ganshuiensis* sp. n., male. **18** Antenna **19** genitalia, lateral view. Scale bar = 0.2 mm.

**Figures 20–21.**
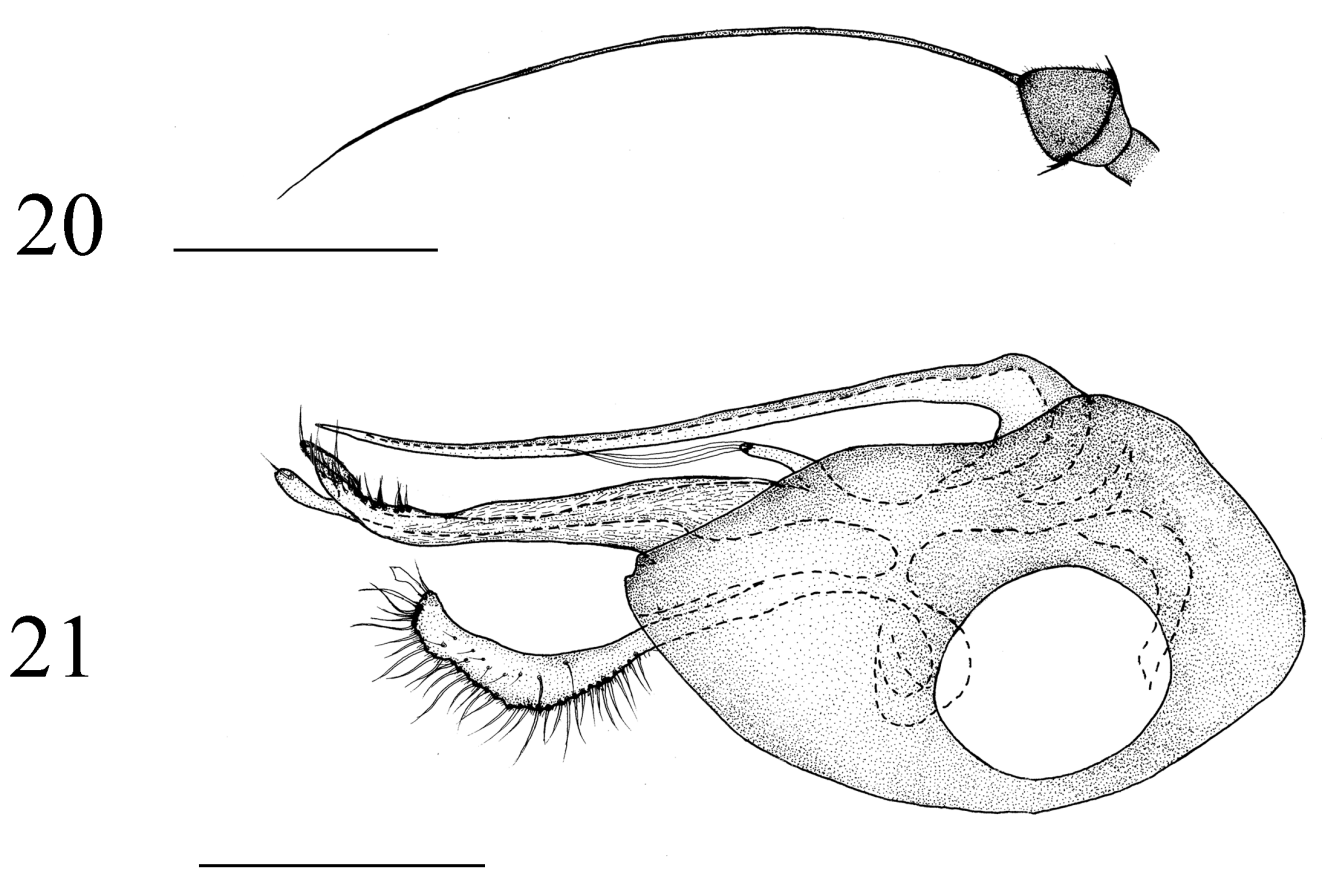
*Medetera lihuae* sp. n., male. **20** Antenna **21** genitalia, lateral view. Scale bar: 0.2 mm.

**Figures 22–23.**
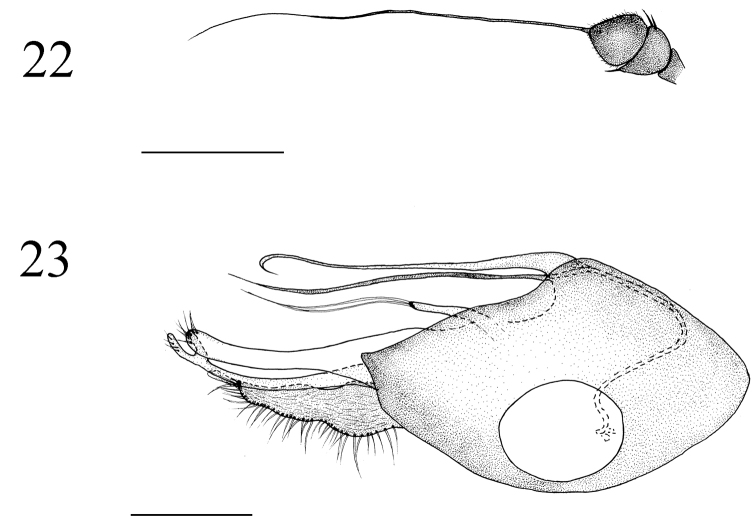
*Medetera shiae* sp. n., male. **22** Antenna **23** genitalia, lateral view. Scale bar = 0.2 mm.

**Figures 24–25.**
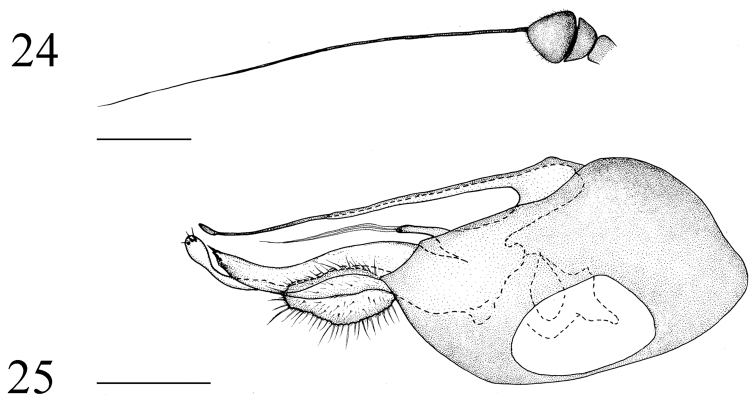
*Medetera shuimogouensis* sp. n., male. **24**. Antenna **25** genitalia, lateral view. Scale bar = 0.2 mm.

**Figures 26–27.**
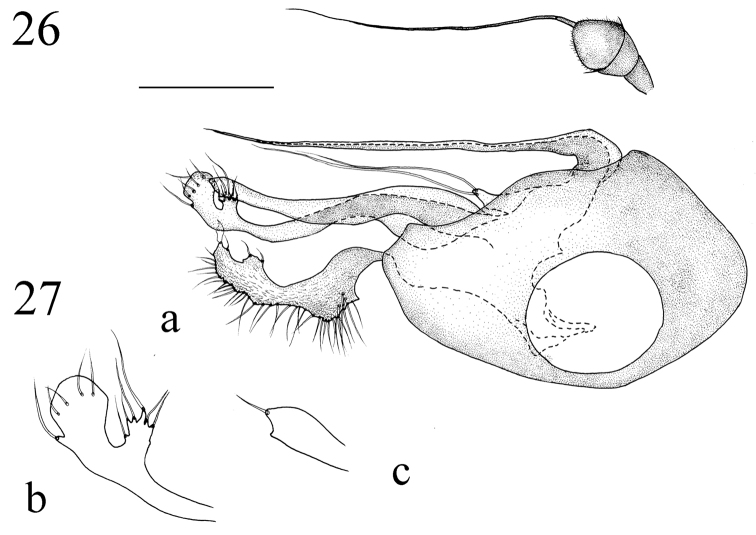
*Medetera transformata* sp. n., male. **26** Antenna **27 a** genitalia, lateral view **b** dorsal surstylus, lateral view **c** ventral surstylus, lateral view. Scale bar: 0.2 mm.

**Figures 28–29.**
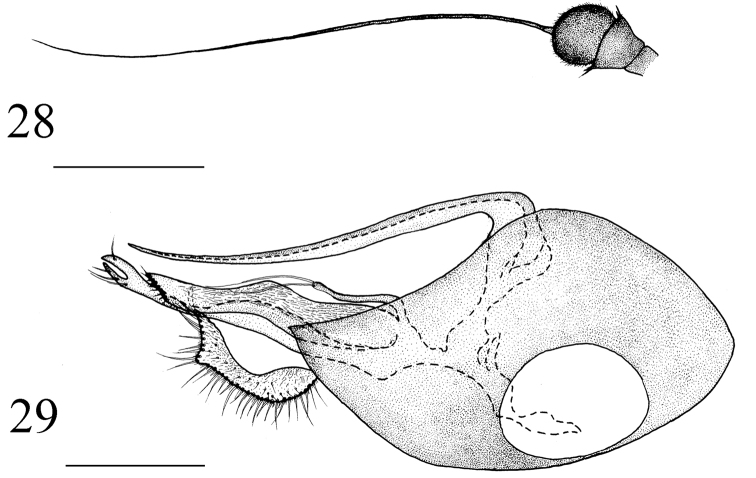
*Medetera xiquegouensis* sp. n., male. **28** Antenna **29** genitalia, lateral view. Scale bar: 0.2 mm.

**Figures 30–39.**
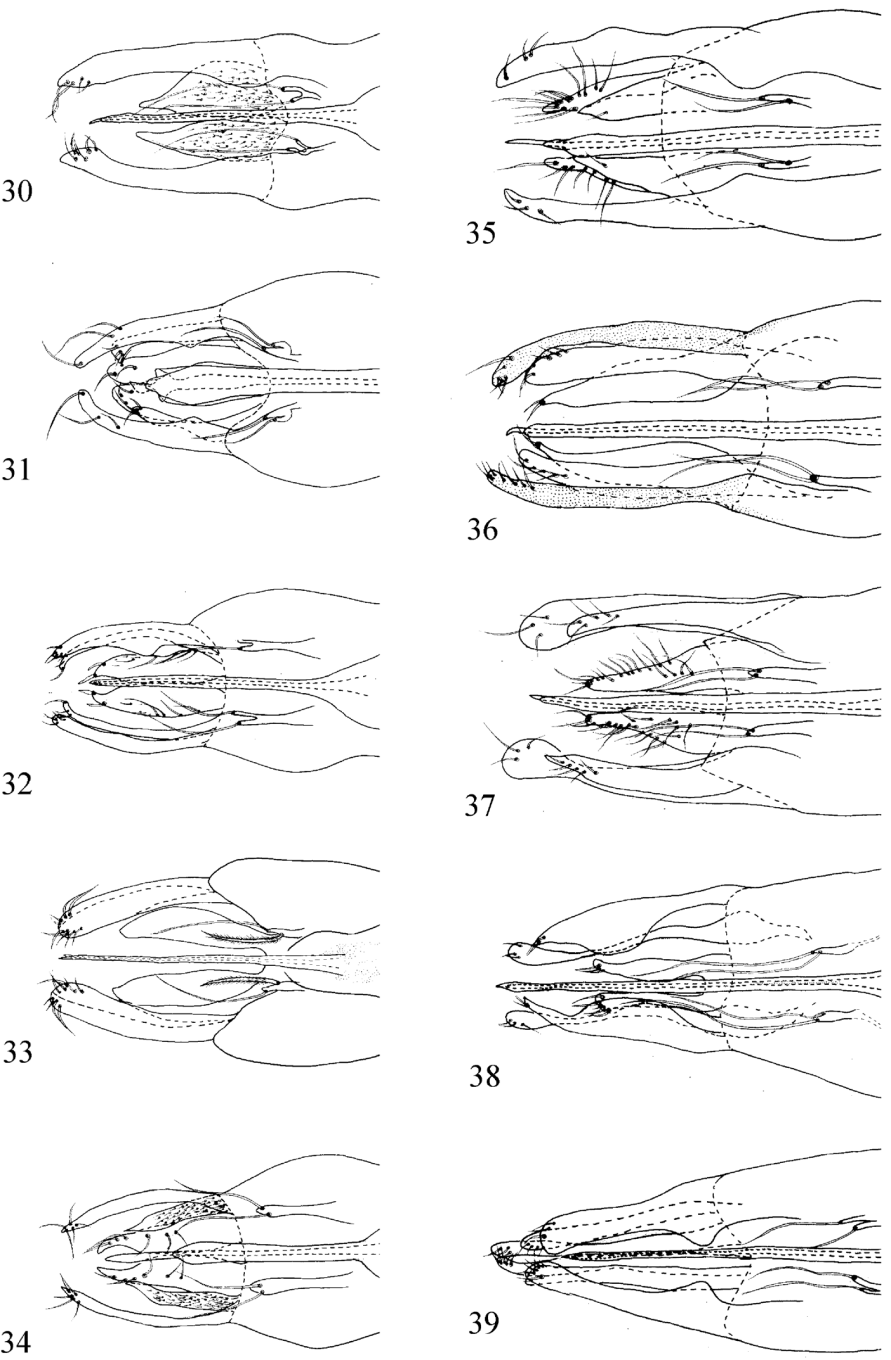
Ventral view of apical half of male genitalia (male). **30** *Medetera albens* sp. n. **31** *Medetera bisetifera* sp. n. **32** *Medetera triseta* sp. n. **33** *Medetera flava* sp. n. **34** *Medetera ganshuiensis* sp. n. **35** *Medetera lihuae* sp. n. **36** *Medetera shiae* sp. n. **37** *Medetera shuimogouensis* sp. n. **38** *Medetera transformata* sp. n. **39** *Medetera xiquegouensis* sp. n. Scale bars: 1 mm.

### *Medetera apicalis* group

The *apicalis* group is not very well defined. Normally the following features are used to distinguish it from the other groups in Palaearctic China: thorax with three long strong dc; epandrium long, nearly twice longer than wide, sometimes with distinct bristle(s); epandrial lobes distinctly separate at base; hypandrium nearly quadrangle, often covering the phallus and holding out at an angle from the epandrium; cercus usually with flattened apicodorsal claw-like bristles. See discussion about the features of the whole group on Bickel (1985: 139).

#### Medetera albens sp. n.

Taxon classificationAnimaliaDipteraDolichopodidae

http://zoobank.org/EA7923D9-EF34-4BBA-9C72-4A749DDD17E2

Figs 1 , 11–12 , 30

##### Diagnosis.

Width of face approximately twice length of first flagellomere. Arista of first flagellomere apical, becoming black to pale yellow from base to tip. Four pairs of dc (anterior one short, posterior three strong). Acr clear and regular. CuAx ratio 0.5. Legs mainly black. Cercus nearly white, strip-like, sharp apically, with one obvious dentation at basal 1/5, five times longer than wide, apical bristle somewhat claw-like.

##### Description.

Male (Fig. 1). Body length 2.2 mm, wing length 2.3 mm. Head: vertex, frons and face dark metallic green with gray pollinosity; eyes separated, face nearly parallel, width of face twice length of first flagellomere. Hairs and bristles on head black except postocular bristles and posteroventral hairs pale yellow. Antenna (Fig. 11) black, first flagellomere nearly triangular, almost as long as wide, shortly brown pubescent; arista apical, becoming black to pale yellow from base to tip, bare, with basal segment extremely short, less than 0.1 times length of apical segment. Proboscis brown with pale white apical hairs; palpus brown with pale white apical hairs.

Thorax metallic green with gray pollinosity. Hairs and bristles on thorax black. Four pairs of dc (anterior one short, posterior three strong), four clear regular biseriate acr at anterior portion, two sa. Scutellum with two pairs of sc (median pair long, strong). Legs becoming dark brown to brown from base to tip onward except tip of femora yellow. Hairs and bristles on legs mainly pale yellow. Fore coxa with six dorsal bristles on apical 1/2; mid coxa with three dorsal bristles and one outer bristle at middle. Hind coxa with one outer bristle at middle. Hind trochanter with one outer bristle at middle. Fore femur with two short ventral hairs apically. Mid femur with four ventral bristles at basal half, gradually becoming longer from base to tip onward. Fore tibia with two short black apical bristles. Hind tibia with five short strong apical spurs. Fore tarsomere one with row of 16 short ventral bristles; hind tarsomere 1 with row of 18 short dorsal and 18 short ventral bristles. Relative length of tibiae and five tarsomeres of legs LI: 2.7 : 1.5 : 0.8 : 0.6 : 0.5 : 0.4; LII: 3.5 : 2.0 : 1.0 : 0.7 : 0.4: 0.4 ; LIII: 4.5 : 1.0 :1.5 : 1.0 : 0.5: 0.5. Wing nearly hyaline, tinged brown; veins brown, R_4+5_ and M_1+2_ convergent apically. CuAx ratio 0.5. Squama pale white with long white hairs. Halter pale yellow.

Abdomen dark metallic green with thin gray pollinosity. Hairs and bristles pale yellow. Male genitalia (Figs 12, 30): Mainly black except epandrial lobes, surstylus and cercus pale to yellow, phallus brown. Hairs and bristles pale yellow. Epandrium longer than wide, epandrial lobes small, fused at base, each with one slender apical bristle. Ventral surstylus long, wide, slightly wide at apex, with three short apical bristles; dorsal surstylus wide and rounded apically, with three short apical bristles. Cercus nearly white, strip-like, narrowed towards tip, with an obvious dentation at basal 1/5; marginal bristles present on weak digitations, five times longer than wide, apical bristle somewhat claw-like. Hypandrium tilted up apically, blunt apically. Phallus thin, hidden within hypandrium.

Female. Unknown.

##### Types.

Holotype male, CHINA, Inner Mongolia, Helan Mountain, Gulaben, Zhonggutian (N39°09'51.29", E106°05'82.66'’), 1892 m altitude, collected by sweeping nets in grass, 2014.VII.13, Yanan Lv (CAU). Paratype: one male, same data as holotype (CAU).

##### Distribution.

Palaearctic: China (Inner Mongolia).

##### Remarks.

This new species is quite unique due to its special arista and the simple shape of the surstylus. It is easily distinguished from the other species of the *Medetera apicalis* group.

##### Etymology.

The specific name refers to the color of the arista, which becomes black to pale yellow gradually from base to tip.

#### Medetera bisetifera sp. n.

Taxon classificationAnimaliaDipteraDolichopodidae

http://zoobank.org/A7CFE77C-B8E2-44EB-8EC3-BDC9EA6B7E3F

Figs 2 , 13–14 , 31

##### Diagnosis.

Width of face about 1.5 times length of first flagellomere. Three pairs of long strong dc in same length, six biseriate acr. Legs mainly yellow, mid femur yellow except narrow blackish dorsal portion. CuAx ratio 0.3. Cercus strip-like, six times longer than wide, ventral margin with three bristles at apical 1/5; tip with two digitations each with one apical bristle. Phallus with distinct preapical lateral wings in ventral view.

##### Description.

Male (Fig. 2). Body length 1.8 mm, wing length 1.5 mm. Head: vertex, frons and face dark metallic green with gray pollinosity; eyes separated, face nearly parallel, width of face approximately 1.5 times length of first flagellomere. Hairs and bristles on head black except postocular bristles and posteroventral hairs pale yellow. Antenna (Fig. 13) black; first flagellomere flat, shortly brown pubescent; arista apical, black, bare, with basal segment extremely short, less than 0.1 times length of apical segment. Proboscis black with black apical hairs; palpus black with black apical bristle.

Thorax metallic green with gray pollinosity. Hairs and bristles on thorax black. Three pairs of long strong dc in same length, six hair-like biseriate acr, two sa. Scutellum with two pairs of sc (median pair long, strong). Legs mainly yellow but coxae, trochanters and all tarsomere 5 black, fore and hind femora dark yellow to yellow except mid femur with narrow blackish portion dorsally. Hairs and bristles on legs mainly pale white. Fore coxa with seven strong anterior bristles; mid coxa with three strong anterior bristles and one outer bristle; hind coxa with one outer bristle at middle and single apical bristle. Mid femur with 12 short dorsal bristles in a row. Mid tibia with one brown paired ad-pd at basal 1/3. Hind tibia with four ventral bristles in apical half and three black apical bristles. Relative length of tibiae and five tarsomeres of legs LI: 2.0 : 1.0 : 0.5 : 0.4 : 0.2 : 0.3; LII: 2.5 : 1.0 : 0.5 : 0.4 : 0.3: 0.3; LIII: 2.5 : 0.6 : 1.0 : 0.5 : 0.3: 0.2. Wing nearly hyaline, tinged brown; veins light brown, R_4+5_ and M_1+2_ convergent apically. M_1+2_ somewhat curve. CuAx ratio 0.3. Squama pale white with short pale hairs. Halter pale yellow.

Abdomen dark metallic green with thin gray pollinosity. Hairs and bristles pale yellow. Male genitalia (Figs 14, 31): Mainly black except epandrial lobes, surstylus and cercus yellow. Hairs and bristles yellow to white. Epandrium longer than wide, epandrial lobes thin finger-like, each with single long thin apical bristle. Ventral surstylus widened towards tip, straight at apex, with row of eight short apical bristles; dorsal surstylus wide and U-shaped apically, straight and thin, ventral lobe with one short apical bristle, dorsal lobe with nine bristles. Cercus strip-like, long, narrowed towards tip, six times longer than wide, base slightly dilated; ventral margin with three bristles at apical 1/5, dorsal margin with dense marginal bristles, tip with two digitations each with one apical bristle. Hypandrium simple. Phallus with distinct preapical lateral wings in ventral view.

Female. Body length 1.9 mm, wing length 1.7 mm. Similar to male except the narrow blackish dorsal portion of mid femur is nearly indistinct.

##### Types.

Holotype male, CHINA, Inner Mongolia, Tongliao, Daqinggou (N42°49'16.6", E122°10'58.0"), 180 m alt., collected by sweeping nets in grass, 2014.VII.6, Ding Yang & Ning Wang (CAU). Paratypes: three females, same data as holotype (CAU).

##### Distribution.

Palaearctic: China (Inner Mongolia).

##### Remarks.

This new species is somewhat similar to *Medetera gussakovskii* Negrobov, 1966 because they both have three dc of same length, they both have two sa, their legs are both mainly yellow and their CuAx ratio are similar (in latter it is 0.32), but can be distinguished from the latter by the color of the antenna and the mid femur as well as the apical bristle of cercus. In *Medetera gussakovskii*, the scape and the pedicel of antenna are yellow and the mid femur is wholly yellow, the cercus has one strong peg-like apical bristle instead of the two digitations with bristles (Negrobov and Stackelberg 1972: p 304, figs 555–557).

##### Etymology.

The specific name refers to the two digitations of the cercus which each has an apical bristle.

#### Medetera triseta sp. n.

Taxon classificationAnimaliaDipteraDolichopodidae

http://zoobank.org/0F5082BC-63AD-4501-A50C-C1A893B4DBF4

Figs 3 , 15 , 32

##### Diagnosis.

Width of face approximately twice length of first flagellomere. Three pairs of long strong dc in same length, four hair-like biseriate acr. CuAx ratio 0.67. Legs mainly black. Ventral surstylus with one external bristle at apical 1/3 on ventral margin, one long apical bristles; dorsal lobe of dorsal surstylus dilated. Cercus strip-like, 2.5 times longer than wide, with blade-like bristles at apex and ventral apical 1/4 point. Phallus and hypandrium both normal in lateral and ventral view.

##### Description.

Male (Fig. 3). Body length 2.0–2.3 mm, wing length 1.75–1.9 mm. Head: vertex, frons and face dark metallic green with gray pollinosity; eyes separated, face nearly parallel, width of face nearly twice length of first flagellomere. Hairs and bristles on head black except postocular bristles and posteroventral hairs pale yellow. Antenna black, first flagellomere oval, 1.2 times longer than wide, shortly brown pubescent; arista apical, black, bare, with basal segment short, nearly 0.1 times length of apical segment. Proboscis black with black apical hairs; palpus black with black apical hairs and one black apical bristle.

Thorax dark metallic green with gray pollinosity. Hairs and bristles on thorax black except pp with three pale white spine-like bristles. Three pairs of long strong dc in same length, four hair-like biseriate acr, two sa. Scutellum with two pairs of sc (median pair long strong). Legs black except apical half of femora, middle 1/3 of fore tibia, mid tibia and main portion of hind tibia, basal half of fore and mid tarsomere 1 yellow. Hairs and bristles on legs mainly pale yellow. Fore coxa with row of 12 short dorsal bristles; mid and hind coxae each with one strong outer bristle at middle and one apical bristle. Mid and hind trochanters each with one spine-like outer bristle at middle. Hind femur with six dorsal bristles and eight short ventral bristles at basal 1/2. Fore tibia with three short apical bristles. Mid tibia with one paired black strong ad-pd at basal 1/5 and two black strong apical bristles. Hind tibia with row of six weak ad at apical 1/3 and two short apical bristles. Hind tarsomere 1 with row of short ventral bristles and two apical bristles. Hind tarsomere 2 with six short av. Relative length of tibiae and five tarsomeres of legs LI: 3.5 : 2.0 : 1.2 : 0.8 : ? : ?; LII: 5.0 : 3.0 : 1.5 : 1.0 : 0.7: 0.6; LIII: 5.0 : 1.3 : 3.0 : 1.3 : 0.5: 0.4 (fore tarsomeres 4-5 missing). Wing nearly hyaline, tinged brown; veins brown, R_4+5_ and M_1+2_ convergent apically. CuAx ratio 0.67. Squama pale white with long pale white hairs. Halter pale yellow.

Abdomen dark metallic green with thick gray pollinosity. Hairs and bristles pale white. Male genitalia (Figs 15, 32): Mainly black except epandrial lobes, surstylus, hypandrium and cercus dark yellow; phallus dark brown. Hairs and bristles yellow to pale white. Epandrium longer than wide; epandrial lobes forming two digitations each with one long thin apical bristle. Ventral surstylus nearly straight, slightly narrower at apical 1/6, blunt at tip, with one external bristle at apical 1/3 on ventral margin, one long and three short apical bristles; dorsal surstylus thin, dilated and U-shaped apically, straight and thin, with ventral lobe thin, sharp at tip, with one long apical bristle, dorsal lobe dilated with three normal apical bristles and one long apical bristle. Cercus strip-like, 2.5 times longer than wide, invaginated at basal 1/3 to apical 1/3 on ventral margin, thick at apical 1/4 to apical 1/3, with dense thin marginal bristles at dorsal margin, one long blade-like apical bristle, one blade-like bristle at apical 1/4 on ventral margin. Hypandrium thin and simple, sharp apically. Phallus thin and normal, hidden within hypandrium.

Female. Unknown.

##### Types.

Holotype male, CHINA, Inner Mongolia, Shuimogou (N49°34'44.8", E125°11'30.1"), 2130 m, collected by sweeping nets in grass, 2014.VII.5, Li Shi (CAU). Paratypes: one male, same data as holotype (CAU); five males, CHINA, Inner Mongolia, keerqinhan, (N46°10'32.8", E122°03'20.5"), 460m, collected by sweeping nets in grass, 2008.VII.19, Gang Yao (CAU); one male, CHINA, Inner Mongolia, Xilinguole (N44°63'69.4", E117°54'35.7"), 1000.4 m, collected by sweeping nets in grass, 2014.VII.13, Yanan Lv (CAU); one male, CHINA, Inner Mongolia, Aergeqihamula (N48°46'42.3", E117°25'21.5"), 598.2 m, collected by sweeping nets in grass, 2014.VII.17, Yanan Lv (CAU).

##### Distribution.

Palaearctic: China (Inner Mongolia).

##### Remarks.

This new species is somewhat similar to *Medetera turkestanica* Stackelberg, 1926 because they both have three strong dc of similar length, their legs are both mainly black, their CuAx ratio are both 0.67 and their face both have gray pollinosity, but can be distinguished from the latter by the color of legs. In *Medetera turkestanica*, legs are all dark metallic green, nearly brown. There is no detailed description of male genitalia of *Medetera turkestanica*, but we are sure they are different species (Negrobov and Stackelberg 1974: 349).

##### Etymology.

The species is named for the three claw-like bristles on the cercus.

### *Medetera diadema*-*veles* group

The *diadema*-*veles* group is one of the most well-defined and characterized group of Medeterinae. For a detailed discussion about the characters used to distinguish the group with other species see Bickel (1985: p 164). The most recognizable feature for species in Palaearctic China would be the epandrial lobes which form one long digitation with two long apical bristles. Species of the group vary on the reduction of hypopygial structures, and mostly differ in the surstylus.

#### Medetera flava sp. n.

Taxon classificationAnimaliaDipteraDolichopodidae

http://zoobank.org/9348B212-23C5-4623-AF98-EDB98B449342

Figs 4 , 16–17 , 33

##### Diagnosis.

Width of face about 1.7 times length of first flagellomere. First flagellomere somewhat rectangular, 0.4 times as long as wide. Four pairs of dc of which posterior three pairs long strong, Five hair-like biseriate acr. Pp with three pale yellow spine-like bristles in equal length. CuAx ratio 0.8. Legs almost entirely yellow. Basolateral bristle of epandrial lobe wholly feather-like. Cercus strip-like, tip depressed at middle, margin on invagination slightly raised at middle, with one short blade-like apical bristle.

##### Description.

Male (Fig. 4). Body length 2.8 mm, wing length 2.5 mm. Head: vertex, frons and face dark metallic green with gray pollinosity; eyes separated, face nearly parallel, width of face about 1.7 times length of first flagellomere. Hairs and bristles on head black except postocular bristles and posteroventral hairs pale yellow. Antenna (Fig. 16) black; first flagellomere somewhat rectangular, 0.4 times as wide as long; arista apical, black and bare, with basal segment extremely short, less than 0.1 times length of apical segment. Proboscis black with pale yellow apical hairs; palpus black with black apical hairs.

Thorax dark metallic green with gray pollinosity. Hairs and bristles on thorax black except pp with three pale yellow spine-like bristles in equal length. Four pairs of dc of which posterior three pairs long strong, five hair-like biseriate acr, two sa. Scutellum with two pairs of sc (median pair long, strong). Legs mainly yellow, but base of fore coxa, mid and hind coxae, extreme base of hind tarsomere 1, apical half of tarsomere 3 and tarsomeres 4–5 black. Hairs and bristles on legs mainly pale yellow. Fore coxa with three apical bristles; mid and hind coxae each with one strong outer bristle at middle and one outer bristle at apical 1/2. Mid femur with six pairs of weak av-pv. Hind femur with six dorsal bristles and eight short ventral bristles, all very thin. Mid tibia with one black ad-pd pair at basal 1/4 and two apical bristles. Hind tibia with eight short ad at apical 1/4 and three apical bristles. Fore and mid tarsomeres 2-4 each with three short black apical bristles. Hind tarsomere 2 with row of 12 short spine-like bristles. Relative length of tibiae and five tarsomeres of legs LI: 2.5 : 1.3 : 1.0 : 0.5 : 0.4 : 0.4; LII: 3.5 : 2.0 : 1.0 : 0.6 : 0.5: 0.3; LIII: 4.0 : 0.8 : 2.0 : 1.0 : 0.5: 0.4. Wing nearly hyaline, tinged brown; veins brown, R_4+5_ and M_1+2_ convergent apically. CuAx ratio 0.8. Squama pale white with long pale hairs. Halter pale yellow.

Abdomen dark metallic green with thick gray pollinosity. Hairs and bristles pale yellow. male genitalia (Figs 17, 33): Mainly black except epandrial lobes, surstylus and cercus brown. Hairs and bristles yellow to pale white. Epandrium longer than wide, epandrial lobes forming one digitation with two long and slender apical bristles, of which basolateral bristle of epandrial lobe wholly feather-like. Ventral surstylus narrowed towards tip, almost straight except one wave near base, tip blunt, with row of six apical bristles; dorsal surstylus wide and U-shaped apically, thin, bent at middle, with one long bristle at apical 1/10, ventral and dorsal lobes each with one short apical bristle. Cercus strip-like, raised at middle on dorsal margin, three times longer than wide; dorsal margin with dense marginal bristles, tip depressed at middle, margin on depression slightly raised at middle, with short dense bristles and one short blade-like bristle apically. Hypandrium simple. Phallus thin, hidden within hypandrium.

Female. Body length 3.0 mm, wing length 2.5 mm. Similar to male.

##### Types.

Holotype male, CHINA, Inner Mongolia, Tumujinur Nur (N46°17'17.6", E122°10'58.0"), 220 m, collected by sweeping nets in grass, 2014.VII.23, Yanan Lv (CAU). Paratypes: 3 females, same data as holotype (CAU).

##### Distribution.

Palaearctic: China (Inner Mongolia).

##### Remarks.

This new species is somewhat similar to *Medetera diadema* Linnaeus, 1767 because they both have black antenna, yellow tibia and one ad, one pd on mid tibia, their bristles on pp are nearly in same length, but can be distinguished from the latter by the CuAx ratio and the color of legs and the bristles on epandrial lobe and cercus. In *Medetera diadema*, the CuAx ratio is 0.56, the coxae and the tarsi are brown, the bristle on epandrial lobe is normal, the cercus has a claw-like apical bristle and one long apical process ventrally (Negrobov and Stackelberg 1972: 296, figs 505–506).

##### Etymology.

The specific name refers to the nearly entirely yellow color of the legs.

#### Medetera ganshuiensis sp. n.

Taxon classificationAnimaliaDipteraDolichopodidae

http://zoobank.orgF2E6F401-63D5-4413-B052-9A8CB7B49B87

Figs 5 , 18–19 , 34

##### Diagnosis.

Width of face about two times length of first flagellomere. Four pairs of dc, anterior two weak and posterior two strong, one biseriate acr. CuAx ratio 1.4. Hind tibia slightly expanded at apex, with five short black apical spurs. Ventral surstylus with two long strong bristles at apical 1/5. Hypandrium narrowed towards tip, sharp apically, thin and simple in lateral and ventral view. Cercus nearly rectangular, two times longer than wide, with one blade-like bristle apically.

##### Description.

Male (Fig. 5). Body length 2.5 mm, wing length 2.5 mm. Head: vertex, frons and face dark metallic green with gray pollinosity; eyes separated, face nearly parallel, width of face about twice length of first flagellomere. Hairs and bristles on head black except postocular bristles black and posteroventral hairs pale yellow. Antenna (Fig. 18) all black; first flagellomere pale white pubescent; arista apical, black, thinly pale white pubescent, nearly bare, with basal segment extremely short, less than 0.1 times length of apical segment. Proboscis dark brown with several radial black strips; palpus black with one brown apical bristles.

Thorax dark metallic green with gray pollinosity. Hairs and bristles on thorax black. Four pairs of dc, anterior two weak and posterior two strong, one biseriate acr at anterior portion, two sa. Scutellum with two pairs of sc (median pair long, strong). Legs mainly black except tip of femora dark yellow and extreme base of mid tibia dark yellow. Hairs and bristles on legs mainly pale white. Fore coxa with one anterior bristle at middle and four ventral apical hairs; mid coxa with two outer bristles at middle; hind coxa with one outer bristle at middle. Hind femur with row of three short av at basal 1/3 to apical 1/3. Fore tibia without distinct bristle; mid tibia with one brown apical bristle; hind tibia slightly expanded at apex, without distinct bristle but with five short black apical spurs. Relative length of tibia and five tarsomeres of legs LI: 3.5 : 1.5 : 1.3 : 1.0 : 0.5 : 0.4; LII: 5.0 : 2.2 : 2.0 : 1.0 : 0.4 : 0.5; LIII: 5.5 : 1.0 : 2.5 : 1.5 : 0.6: 0.6. Wing nearly hyaline, tinged brown; veins brown, R_4+5_ and M_1+2_ convergent apically. CuAx ratio 1.4. Squama pale white with pale hairs. Halter pale yellow.

Abdomen dark metallic green with thin gray pollinosity. Hairs and bristles pale yellow. Tergite 2 with one circulate of bristles apically. Male genitalia (Figs 19, 34): Mainly black. Hairs and bristles mainly pale white except two long strong bristles at apical 1/5 of ventral surstylus black. Epandrium longer than wide; epandrial lobes forming one digitation with two long slender apical bristles. Ventral surstylus long, narrowed towards tip, almost straight, dilated at basal 1/2, with two long strong bristles at apical 1/5 portion (one bristle at apical 1/8, one bristle at apical 1/10); dorsal surstylus thin, slightly narrowed towards tip, wide and U–shaped apically, dorsal lobe with three short weak bristles, ventral lobe with one long, straight bristle at tip; lobes both sharp at apex. Cercus nearly rectangular, somewhat wave-like at base, nearly two times longer than wide, with one blade-like bristle apically; covered with thin bristles, but marginal bristles distributed averagely. Hypandrium narrowed towards tip, sharp apically, thin and simple in lateral and ventral view. Phallus hidden in hypandrium.

Female. Unknown.

##### Types.

Holotype male, CHINA, Inner Mongolia, Helan Mountain, Gulaben, Ganshu Bay (N38°59.165', E106°02.255'), 2300 m, collected by sweeping nets in grass, 2010.VIII.9, Lihua Wang (CAU). Paratype: one male, same data as holotype (CAU).

##### Distribution.

Palaearctic: China (Inner Mongolia).

##### Remarks.

This new species is somewhat similar to *Medetera mongolica* Negrobov, 1966 because their legs are both mainly black, they both almost do not have acr and their cercus both have one strong apical bristle, but can be distinguished from the latter by the bristle of surstylus and the shape of cercus. In *Medetera mongolica*, one of the apical bristles of dorsal surstylus is flagellate and the cercus has a ventral process (Negrobov and Stackelberg 1972: p 319, figs 669–672).

##### Etymology.

The species is named for the type locality, Ganshu.

#### Medetera lihuae sp. n.

Taxon classificationAnimaliaDipteraDolichopodidae

http://zoobank.org/ECF80C42-DED0-474E-A457-949F9B407839

Figs 6 , 20–21 , 35

##### Diagnosis.

Width of face about 1.5 times length of first flagellomere. Four pairs of dc in same length, four uniseriate acr. Vein M_1+2_ bent. CuAx ratio 1.0. Ventral surstylus long, wide, slightly narrow at apex, with row of ten ventral bristles at apical 1/4 and six thin apical bristles; dorsal surstylus wide and U-shaped apically, ventral lobe with row of six bristles, dorsal lobe with one preapical bristle. Cercus nearly rectangular, with one blade-like apical bristle, six times longer than wide.

##### Description.

Male (Fig. 6). Body length 2.1 mm, wing length 2.3 mm. Head: vertex, frons and face dark metallic green with gray pollinosity; eyes separated, face nearly parallel, width of face about 1.5 times length of first flagellomere. Hairs and bristles on head black except postocular bristles and posteroventral hairs pale yellow. Antenna (Fig. 20) black; first flagellomere nearly triangular, nearly as long as wide, with shortly brown pubescent; arista apical, black, bare, with basal segment extremely short, less than 0.1 times length of apical segment. Proboscis brown with pale white apical hairs; palpus wide, brown with 1 pale white apical bristle.

Thorax dark metallic green with gray pollinosity. Hairs and bristles on thorax black. Four pairs of dc in same length, four uniserate acr, two sa. Scutellum with two pairs of sc (median pair long strong). Legs all black except tips of mid and hind femora dark yellow. Hairs and bristles on legs mainly pale yellow. Fore coxa with six anterior bristles; mid and hind coxae each with one outer bristle at middle. Hind trochanter with one outer bristle at middle. Mid femur with six ventral bristles at basal half, of which middle three relatively long. Hind tibia expanded apically, with three short black apical bristles. Fore tarsomere 1 with row of 12 short ventral bristles; hind tarsomere 1 with row of 12 short dorsal and 12 short ventral bristles. Relative length of tibiae and five tarsomeres of legs LI: 2.7 : 1.2 : 1.0 : 0.7 : 0.4 : 0.3; LII: 3.7 : 2.5 : 1.0 : ? : ?: ?; LIII: 4.0 : 1.0 :1.8 : 1.0 : 0.5: 0.5 (mid tarsomeres 3-5 missing). Wing nearly hyaline, tinged brown; veins brown, R_4+5_ and M_1+2_ convergent apically. M_1+2_ somewhat bent. CuAx ratio 1.0. Squama pale white with short pale white hairs. Halter pale yellow.

Abdomen dark metallic green with thin gray pollinosity. Hairs and bristles pale yellow. Male genitalia (Figs 21, 35): Mainly black except epandrial lobes, surstylus and cercus dark yellow to brown, phallus dark brown. Hairs and bristles yellow to pale white. Epandrium longer than wide; epandrial lobes forming one long digitation with two long thin apical bristles. Ventral surstylus long, wide, slightly narrow at apex, with row of ten ventral bristles at apical 1/4 and six thin apical bristles; dorsal surstylus wide and U-shaped apically, ventral lobe with row of six bristles, dorsal lobe with one preapical bristle. Cercus nearly rectangular, with one blade-like apical bristle, with long marginal bristles and external bristles, six times longer than wide. Hypandrium simple. Phallus thin, hidden within hypandrium.

Female. Unknown.

##### Types.

Holotype male, CHINA, Inner Mongolia, Helan Mountain, Shuimogou (N38°96'36.29", E105°85'78.64"), 1300 m, collected by sweeping nets in grass, 2010.VIII.4, Lihua Wang (CAU). Paratype: one male, same data as holotype (CAU).

##### Distribution.

Palaearctic: China (Inner Mongolia).

##### Remarks.

This new species is somewhat similar to *Medetera ussuriana* Negrobov, 1977 because they both have legs which are mainly black, they both have four strong dc of the same length and their CuAx ratio are both 1.0, but can be distinguished from the color and bristles of/on legs and the bristles on cercus. In *Medetera ussuriana*, the legs are all black except the trochanters yellow, the mid tibia has a pd and the cercus has one claw-like apical bristle (Negrobov and Stackelberg 1977: p 350–351).

##### Etymology.

The species is named after the collector Lihua Wang.

#### Medetera shiae sp. n.

Taxon classificationAnimaliaDipteraDolichopodidae

http://zoobank.org/9379B921-5DCB-4C8D-B953-F2D214F77B1A

Figs 7 , 22–23 , 36

##### Diagnosis.

Width of face about 1.5 times length of first flagellomere. First flagellomere rounded, nearly 0.8 times longer than wide. Four pairs of dc in same length, eight biseriate acr. M-Cu somewhat curved. CuAx ratio 0.61. Cercus strip-like, narrowed towards tip, base dilated, with one apically dilated blade-like apical bristle. Hypandrium thin, tilted back at tip. Phallus thin and sharp at tip, almost totally separate from hypandrium.

##### Description.

Male (Fig. 7). Body length 3.0 mm, wing length 2.5 mm. Head: vertex, frons and face dark metallic green with gray pollinosity; eyes separated, face nearly parallel, width of face about 1.5 times length of first flagellomere. Hairs and bristles on head black except postocular bristles and posteroventral hairs pale yellow. Antenna (Fig. 22) black; first flagellomere rounded, nearly 0.8 times longer than wide, shortly brown pubescent; arista apical, black, bare, with basal segment extremely short, less than 0.1 times length of apical segment. Proboscis extremely wide, black with pale white apical hairs; palpus black with pale white apical bristle.

Thorax metallic green with gray pollinosity. Hairs and bristles on thorax black. Four pairs of dc in same length, eight biseriate acr, two sa. Scutellum with two pairs of sc, median pair long strong. Legs all black, but brown to yellow at apical 1/3 of femora, dark yellow at tibiae except extreme base and tip, yellow at apical half of tarsomere 1. Hairs and bristles on legs mainly black, but pale yellow on tarsus. Fore coxa with four ventral bristles at apical half; mid and hind coxae each with one outer bristle at middle. Fore trochanter with two bristles at middle. Hind tibia with two short apical bristles. Relative length of tibiae and five tarsomeres of legs LI: 3.0 : 1.3 : 1.0 : 0.6 : 0.3 : 0.3; LII: 4.5 : 2.2 : 1.3 : 0.7 : 0.5: 0.5 ; LIII :5.0 : 1.2 :2.1 : 1.2 : 0.5: 0.5. Wing nearly hyaline, tinged brown; veins brown, R_4+5_ and M_1+2_ convergent apically. M-Cu somewhat curved. CuAx ratio 0.61. Squama pale white with long pale white hairs. Halter pale yellow.

Abdomen dark metallic green with thin gray pollinosity. Hairs and bristles pale yellow. Male genitalia (Figs 23, 36) Mainly black except epandrial lobes, surstylus and cercus dark yellow, phallus black. Hairs and bristles yellow to pale white. Epandrium longer than wide; epandrial lobes forming one long digitation with two long and slender apical bristles. Ventral surstylus straight, round at apex, with row of six long apical bristles; dorsal surstylus wide and U-shaped apically, straight and thin, ventral lobe with one preapical long spine-like bristle, dorsal lobe with four short bristles. Cercus strip-like, three times longer than the widest point, narrowed towards tip, base dilated, ventral and dorsal margins each invaginated at basal 1/3 and dorsal 1/3, with long and dense marginal bristles at dorsal margin, with one apically dilated blade-like apical bristle. Hypandrium thin, tilted back at tip. Phallus thin and sharp at tip, almost entirely separate with hypandrium.

Female. Unknown.

##### Types.

Holotype male, CHINA, Inner Mongolia, Helan Mountain, fork of the main peak (N38°51'02.5", E105°49'09.4"), 2247 m, collected by sweeping nets in grass, 2014.VII.6, Li Shi (CAU). Paratypes: three males, same data as holotype (CAU).

##### Distribution.

Palaearctic: China (Inner Mongolia).

##### Remarks.

This new species is somewhat similar to *Medetera paralamprostoma* Negrobov, 1974 because they share the similarity in dc and the color of legs and the size, but can be distinguished from the latter by the bristles on legs, the CuAx ratio and the shape of hypandrium. In *Medetera paralamprostoma*, the CuAx ratio is 1.625, hind femur has one long ad at base and hind tibia has one long preapical pd. The hypandrium is thin and wedge-like (Negrobov and Stackelberg 1974: 327).

##### Etymology.

The species is named after the collector Li Shi.

#### Medetera shuimogouensis sp. n.

Taxon classificationAnimaliaDipteraDolichopodidae

http://zoobank.org/247B6A5F-A8BC-40E2-B879-73A32A753439

Figs 8 , 24–25 , 37

##### Diagnosis.

Width of face approximately twice length of first flagellomere. Four pairs of dc (anterior one short, posterior three strong), four biseriate acr. CuAx ratio 0.8. Phallus thin with big and round apex. Ventral surstylus long, wide, slightly narrowed towards tip, with three short apical bristles; dorsal surstylus thin, narrowed towards tip, wide and rounded apically, with three short apical bristles. Cercus nearly oval, 1.8 times longer than wide, without specialized bristles, covered with thin bristles (marginal bristles distributed averagely).

##### Description.

Male (Fig. 8). Body length 2.4–2.5 mm, wing length 3.5 mm. Head: vertex, frons and face metallic green with gray pollinosity; eyes separated, face nearly parallel, width of face about twice length of first flagellomere. Hairs and bristles on head black except postocular bristles black and posteroventral pale yellow. Antenna (Fig. 24) all black; first flagellomere 0.8 times longer than wide; arista apical, thinly black pubescent, nearly bare, with basal segment extremely short, less than 0.1 times length of apical segment. Proboscis dark brown with radius dark brown strips, and with short pale apical hairs; palpus black with one strong black apical bristles.

Thorax metallic green with gray pollinosity. Hairs and bristles on thorax dark yellow. Four pairs of dc (anterior one short, posterior three strong), four regular biseriate acr at anterior portion, two sa, pp with two yellow bristles not in equal length. Scutellum with two pairs of sc (median pair long strong). Legs black except tips of femora yellow. Hairs and bristles on legs mainly pale. Fore coxa with two rows of four paired ad-pd at apical 1/2; mid coxa with two outer bristles at middles; hind coxa with one outer bristle at middle. Mid trochanter with three short bristles apically. Hind femur with two rows of four short paired ad-pd at basal 1/3. Fore tibia with three short ventral apical bristles. Mid tibia with one brown ad at basal 2/5, one pd at basal 1/10 and two short ventral apical bristles; hind tibia without distinct bristles. Relative length of tibia and five tarsomeres of legs LI: 5.0 : 1.0 : 1.5 : 1.0 : 0.7 : 0.5; LII: 6.5 : 3.0 : 2.0 : 1.6 : 0.6 : 0.7; LIII: 7.5 : 1.5 : 4.0 : 1.6 : 0.8: 0.7. Wing nearly hyaline, tinged brown; veins brown, R_4+5_ and M_1+2_ convergent apically. CuAx ratio 0.8. Squama pale white with pale white hairs. Halter pale yellow.

Abdomen metallic green with thin gray pollinosity. Hairs and bristles pale yellow. Male genitalia (Figs 25, 37): Mainly black except epandrial lobes, surstylus and cercus dark yellow, phallus dark brown. Hairs and bristles yellow to pale white. Epandrium longer than wide; epandrial lobes forming one digitation with two slender apical bristles. Ventral surstylus long, wide, slightly narrowed towards tip, with three short apical bristles; dorsal surstylus thin, narrowed towards tip, wide and rounded apically, with three short apical bristles. Cercus nearly oval, 1.8 times longer than wide, without specialized bristles, covered with thin bristles (marginal bristles distributed averagely). Hypandrium narrowed towards tip, blunt apically, thin and simple in lateral view. Phallus thin with big and round apex.

Female. Unknown.

##### Types.

Holotype male, CHINA, Inner Mongolia, Helan Mountain, Shuimogou (N38°96'42.40", E105°85'82.60'’), 1270 m, collected by sweeping nets in grass, 2010.VIII.6, Yan Li (CAU). Paratypes: two males, same data as holotype (CAU).

##### Distribution.

Palaearctic: China (Inner Mongolia).

##### Remarks.

This new species is unique for the cercus as it has no obvious bristle. The other parts of the species are somewhat like *Medetera feminina* Negrobov, 1967 as they have similar dc and the bristles on pp, the halter and the color and the bristles of/on legs, but can be distinguished from the latter by the shape of phallus, in *Medetera feminina*, the phallus is curved like the beak of an eagle apically. (Negrobov and Stackelberg 1972: 299, figs 527–529, 535).

##### Etymology.

The species is named for the type locality, Shuimogou.

#### Medetera transformata sp. n.

Taxon classificationAnimaliaDipteraDolichopodidae

http://zoobank.org/2B4F6BD4-4DC9-4898-B4B5-243E4539413F

Figs 9 , 26–27 , 38

##### Diagnosis.

Width of face about 2.5 times length of first flagellomere. Four pairs of long strong spine-like dc, seven short weak hair-like biseriate acr. CuAx ratio 1.0. Ventral surstylus nearly straight, wide at basal 1/3, sharp apically, with a small preapical protuberance, with one spine-like apical bristle; dorsal surstylus thin, but dilated and U-shaped apically. Cercus strip-like, with dense marginal bristles and three long external bristles at basal 1/4. Hypandrium slightly expanded preapically in ventral view.

##### Description.

Male (Fig. 9). Body length 2.5 mm, wing length 2.2 mm. Head: vertex, frons and face dark metallic green with gray pollinosity; eyes separated, face nearly parallel, width of face about 2.5 times length of first flagellomere. Hairs and bristles on head black except postocular bristles and posteroventral hairs pale yellow. Antenna (Fig. 26) black; first flagellomere nearly triangular, 1.1 times longer than wide, blunt at tip, shortly brown pubescent; arista apical, black, bare, basal segment short, nearly than 0.15 times length of apical segment. Proboscis black with black apical hairs; palpus black with black apical bristle.

Thorax metallic green with gray pollinosity. Hairs and bristles on thorax black except pp with three pale yellow spine-like bristles in same length. Four pairs of long strong spine-like dc, seven short weak hair-like biseriate acr, two sa. Scutellum with two pairs of sc (median pair long strong). Legs black except trochanters, tip of femora, mid tibia and basal half of mid tarsomere 1 yellow; hind tibia brown. Hairs and bristles on legs mainly pale yellow. Fore coxa with some short dorsal hairs and five-six short apical bristles; mid coxa with one strong outer bristle at middle and one relatively weak bristle at apical 1/3, hind coxa with one outer bristle at middle. Hind femur with five dorsal bristles and six short ventral bristles at basal 1/2. Fore tibia with two short brown apical bristles. Mid tibia with paired black strong ad-pd at basal 1/3 and two black strong apical bristles. Hind tibia with paired ad-pd at apical 1/6 and two short apical bristles. Fore tarsomere 1 with row of short ventral bristles. Hind tarsomere 1 with four short and thick black ventral bristles. Relative length of tibiae and five tarsomeres of legs LI: 2.5 : 1.0 : 0.8 : 0.5 : 0.3 : 0.4; LII: 3.5 : 2.0 : 1.0 : 0.7 : 0.3: 0.4; LIII: :3.5 : 0.5 : 1.7 : ? : ?: ? (hind tarsomeres 3-5 missing). Wing nearly hyaline, tinged brown; veins brown, R_4+5_ and M_1+2_ convergent apically. CuAx ratio 1.0. Squama pale white with long pale white hairs. Halter pale yellow.

Abdomen dark metallic green with thick gray pollinosity. Hairs and bristles pale yellow. Male genitalia (Fig. 22): Mainly black except epandrial lobes, surstylus and hypandrium dark yellow; cercus and phallus dark brown. Hairs and bristles yellow to pale white. Epandrium longer than wide; epandrial lobes forming one digitation with two long thin apical bristles. Ventral surstylus nearly straight, wide at basal 1/3, sharp apically, with a small preapical protuberance, with one spine-like apical bristle; dorsal surstylus thin, but dilated and U-shaped apically, ventral lobe short and wide, with six digitations each with one apical bristle, dorsal lobe dilated with one small digitation bearing one long apical bristles at basal 1/3 on dorsal margin and four preapical external bristles. Cercus strip-like, thick at basal 1/4 to middle on dorsal margin and apical 1/8 to 1/4 on ventral margin, with dense marginal bristles and three long external bristles at basal 1/4, two short bristles on the thick part of ventral margin and one short preapical bristle on ventral margin, and one apical blade-like bristle. Hypandrium thin and simple, sharp apically, slightly expanded preapically in ventral view. Phallus thin, hidden within hypandrium.

Female. Unknown.

##### Types.

Holotype male, CHINA, Inner Mongolia, Shuimogou (N49°34'44.8", E125°11'30.1"), 2130 m, collected by sweeping nets in grass, 2014.VII.5, Li Shi (CAU). Paratypes: two males, CHINA, Inner Mongolia, Helan Mountain, Xiangchizigou (N38°59'60.2", E105°70'23.0"), 1950 m, collected by sweeping nets in grass, 2013.VIII.30, Xiao Zhang (CAU); two males, CHINA, Inner Mongolia, Helan Mountain, Xilinguole, Dongwuqi (N46°23'42.8", E118°48'28.7"), 870 m, collected by sweeping nets in grass, 2014.VII.14, Yanan Lv (CAU).

##### Distribution.

Palaearctic: China (Inner Mongolia).

##### Remarks.

This new species is unique for the shape of the dorsal surstylus and easily separated from other known species. It is somewhat like *Medetera murina* Becker, 1917 as they have similar dc, the arista, the bristles on mid tibia, the color of legs and the CuAx ratio; but can be distinguished from the latter by the number of acr, the bristles on fore and mid femora and the shape of cercus. In *Medetera murina*, thorax has four-five pairs of acr, fore and mid femora each has one av, and the cercus has a deep apical incision. (Negrobov and Stackelberg 1974: p 321–322, figs 693–695).

##### Etymology.

The species is named for the shape of the dorsal surstylus.

#### Medetera xiquegouensis sp. n.

Taxon classificationAnimaliaDipteraDolichopodidae

http://zoobank.org/7C7FDB69-EFEA-4872-A28B-BC1D8DA4C794

Figs 10 , 28–29 , 39

##### Diagnosis.

Width of face about 1.5 times length of first flagellomere. Six biseriate acr. CuAx ratio 1.25. Ventral surstylus slightly waved at middle, straight at apex, dorsal surstylus wide and U-shaped apically, dilated at basal 1/4, thin at middle to apical 1/4, ventral lobe with one preapical bristle, dorsal lobe with three bristles. Cercus strip-like, without specialized bristle, six times longer than wide.

##### Description.

Male (Fig. 10). Body length 2.6 mm, wing length 2.5 mm. Head: vertex, frons and face dark metallic green with gray pollinosity; eyes separated, face nearly parallel, width of face about 1.5 times length of first flagellomere. Hairs and bristles on head black except postocular bristles and posteroventral hairs pale yellow. Antenna (Fig. 28) black, first flagellomere rounded, shortly brown pubescent; arista apical, black, bare, with basal segment extremely short, less than 0.1 times length of apical segment. Proboscis black with black apical hairs; palpus black with one black apical bristle.

Thorax metallic green with gray pollinosity, two sa. Hairs and bristles on thorax black. With five pairs of dc, six biseriate acr, two sa. Scutellum with two pairs of sc (median long, strong). Legs all black. Hairs and bristles on legs mainly pale yellow. Fore coxa with eight dorsal bristles at apical 1/2; mid and hind coxae each with one outer bristle at middle. Mid femur with four ventral bristles at basal 1/4 to apical 1/4. Hind tibia with four short dorsal bristles at apical 1/5, expanded apically, with five short apical bristles. Relative length of tibiae and five tarsomeres of legs LI: 3.0 : 1.3 : 1.0 : 0.6 : 0.3 : 0.3; LII: 4.5 : 2.2 : 1.3 : 0.7 : 0.5: 0.5; LIII: 5.0 : 1.2 :2.1 : 1.2 : 0.5: 0.5. Wing nearly hyaline, tinged brown; veins brown, R_4+5_ and M_1+2_ convergent apically. R_4+5_ and M_1+2_ arched towards R_2+3_. CuAx ratio 1.25. Squama pale white with short pale white hairs. Halter pale yellow.

Abdomen dark metallic green with thin gray pollinosity. Hairs and bristles pale yellow. Male genitalia (Figs 29, 39): Mainly black except epandrial lobes, surstylus, cercus and phallus brown. Hairs and bristles yellow to pale white. Epandrium longer than wide; epandrial lobes forming one long digitation with two slender apical bristles. Ventral surstylus slightly waved at middle, straight at apex, with row of eight short apical bristles; dorsal surstylus wide and U-shaped apically, dilated at basal 1/4, thin at middle to apical 1/4, ventral lobe with one preapical bristle, dorsal lobe with three bristles. Cercus strip-like, somewhat invaginated at ventral margin, with a dentation at apical 2/5 on dorsal margin, narrower at tip, without specialized bristle, with long bristles at dorsal margin and short external bristles, six times longer than wide. Hypandrium simple. Phallus thin, hidden within hypandrium.

Female. Unknown.

##### Types.

Holotype male, CHINA, Inner Mongolia, Helan Mountain, Xiquekou of Yao Bay (N38°57'57.5", E105°50'52.0"), 1340 m, collected by sweeping nets in grass, 2010.VIII.1,Yan Li (CAU). Paratype: one male, same data as holotype (CAU).

##### Distribution.

Palaearctic: China (Inner Mongolia).

##### Remarks.

This new species is somewhat similar to *Medetera infuscata*, 1974 Negrobov because their legs are both mainly black and the shape of their cercus and hypandrium are quite similar, their dc and acr are similar in size but differ in amount, the new species can be distinguished from the latter by the CuAx ratio and the shape of phallus. In *Medetera infuscata*, the CuAx ratio is 0.75 and the phallus is apically hooked (Negrobov and Stackelberg 1974: p 307, figs 580–584).

##### Etymology.

The species is named for the type locality, Xiquekou.

## Supplementary Material

XML Treatment for Medetera albens

XML Treatment for Medetera bisetifera

XML Treatment for Medetera triseta

XML Treatment for Medetera flava

XML Treatment for Medetera ganshuiensis

XML Treatment for Medetera lihuae

XML Treatment for Medetera shiae

XML Treatment for Medetera shuimogouensis

XML Treatment for Medetera transformata

XML Treatment for Medetera xiquegouensis
